# CNS relapse of diffuse large B cell Lymphoma A single centre experience

**DOI:** 10.12669/pjms.336.13812

**Published:** 2017

**Authors:** Adil Nazir, Fawad, Neelam Siddique, Abdul Hameed

**Affiliations:** 1 Dr. Adil Nazir, MBBS, FCPS (Medicine). Fellow Medical Oncology, Department of Medical Oncology, Shaukat Khanum Memorial Cancer Hospital & Research Centre, Lahore, Pakistan; 2 Dr. Fawad, MBBS, FCPS (Medicine). Fellow Medical Oncology, Department of Medical Oncology, Shaukat Khanum Memorial Cancer Hospital & Research Centre, Lahore, Pakistan; 3 Dr. Neelam Siddiqui, MBBS, FRCP, CCST (Medical Oncology). Consultant medical Oncologist, Department of Medical Oncology, Shaukat Khanum Memorial Cancer Hospital & Research Centre, Lahore, Pakistan; 4 Dr. Abdul Hameed, MBBS, MD, FRCP (Edin).Consultant Hematologist, Department of Medical Oncology, Shaukat Khanum Memorial Cancer Hospital & Research Centre, Lahore, Pakistan

**Keywords:** CNS relapse, Chemotherapy, Survival, Prophylaxis

## Abstract

**Background and Objective:**

Central nervous system (CNS) relapse of diffuse large B cell lymphoma (DLBCL) is relatively uncommon and nearly fatal. Two years CNS relapse risk is 0.8% in low, 3.9% in intermediate and 12% in high risk patients. Our aim was to study, the baseline characteristics and outcome in term of median survival of DLBCL patients with CNS relapse.

**Methods:**

This is a retrospective analysis. All patients of DLBCL with CNS relapse from 2006 to 2014 were included. Data were collected from computerized Hospital Information System and analyzed for characteristics and median survival.

**Results:**

Out of twenty one patients included in the study, 14(66.3%) males and 7(33.7%) were females. On initial diagnosis of DLBCL, median age was 37.4 years (27-47). Ann Arbor stage of I-IV was in 3 (14.3%), 2(9.5%), 4(19%) and 12(57.1%) patients, respectively. Extra-nodal involvement was noted in 16(76.2%), high LDH in 18(85.7%), bone marrow involvement in 8(38.1%) and bulky disease in 5(23.8%) patients. International Prognostic Index (IPI) score was 1 in 4(19%), 2 in 9(42.9%), 3 in 8(38.1%) patients. Extra-nodal sites were gut in 2(9.1%) while 1(4.5%) patient of each of following organs involvement was seen: cervix, gluteal muscle, iliac bone, liver, ovaries, pancreas, parotid gland and testes. Chemotherapy CHOP was given to 16(76.2%) and RCHOP in 5(23.8%) patients. Prophylactic intrathecal methotrexate was given to 10(47.6%) patients. Complete response was in 10 (47.6%), partial response was in 3 (14.3%) and disease progression was in 8 (38.1%) patients. CNS relapse occurred in 17 (81%) patients within six months after completion of therapy. CNS relapse along with systemic disease was in 14(66.6%) patients. Isolated CNS relapse was noted in 7(33.3%) patients. Second line chemotherapy regimens were HDMTX 5(23.8%), HDMTX/TRIO IT 3(14.2%), HDMTX/HDAC 2(9.5%), HCVAD 3(14.2%), ICE 4(19.4%), DHAP 1(4.7%), ICE/HDMTX 1(4.7%), none 2(9.5%). Overall median survival of CNS relapsed patients was 54 days.

**Conclusion:**

Patients with DLBCL who had advanced stage, high LDH and extra-nodal involvement at initial presentation are at high risk for CNS relapse. About half of the patients had CNS relapse despite primary CNS prophylaxis. Once relapsed in CNS, these patients have very poor prognosis.

## INTRODUCTION

Diffuse large B-cell lymphoma (DLBCL) is the most common lymphoid neoplasms in adults; accounting for approximately 32.5% of NHLs diagnosed annually.^1^ Secondary central nervous system (CNS) involvement in DLBCL includes an isolated CNS relapse or CNS involvement with systemic disease. This rare but fatal clinical problem is a therapeutic dilemma in the management of DLBCL.^2^ In DLBCL, the incidence of CNS relapse is 2.2% to 5% and 1.6% as isolated CNS relapse. Most relapses occur with a median duration of less than one year from the time of initial diagnosis.^3^-^6^ There is association of increased risk for developing CNS relapse in patients with elevated LDH, ≥2 extranodal sites and involvement of specific extranodal sites (testes, paranasal sinus, breast and bone marrow) on initial presentation.^7^-^9^ There is a validated prognostic model to predict the risk of CNS relapse utilizing five clinical factors (age > 60 years, LDH > normal, stage III or IV, ECOG PS >1, and involvement of the kidney or adrenal gland) thus recommending for CNS prophylaxis therapy.^10^,^11^ Risk of CNS relapse is higher in activated B-cell (ABC) than germinal center B-cell (GCB).^12^,^13^ Addition of rituximab to chemotherapy (CHOP) may improve the remission rate and overall survival of patients with DLBCL. However, question about risk reduction of CNS relapse with rituximab still remains unanswered with mixed results.^14^ There is no strong evidence that supports any single approach for CNS prophylaxis.^15^ Intrathecal methotrexate given at least once per systemic treatment cycle has been used for many years however, high-dose IV methotrexate-based prophylaxis may lower incidence of CNS relapses.^16^-^19^

Aim of this study was to reveal the baseline characteristics and risk factors associated with CNS relapse in patients with DLBCL. We also looked at the overall survival of these patients and impact of intrathecal chemotherapy.

## METHODS

This is a retrospective analysis of total DLBCL twenty one patients treated at our institute. All patients of DLBCL with CNS relapse from 2006 to 2014 were included. Data were collected from Hospital Information System and analyzed for characteristics and median survival.

### Statistical analysis

The data were analyzed using Statistical Package for Social Sciences (SPSS) version 19. Descriptive statistics were performed. Categorical data such as gender, LDH, extra nodal site involvement, bone marrow Involvement, bulky disease, IPI score, stage of disease, chemotherapy, response to chemotherapy were presented as frequency and percentage. Quantitative variables including age were mentioned as mean ± standard deviation. Base line characteristics were compared by using chi square test. Survival was measured by Kaplan-Meier curves. P-value less than 0.05 were considered significant.

## RESULTS

Out of twenty one patients included in the study, 14(66.3%) males and 7(33.7%) were females. On initial diagnosis of DLBCL, median age was 37.4 years (27-47). Stage IV was in 12(57.1%) patients; whereas 4(19.0%), 2(9.5%) and 3 (14.3%) patients had stage III, II, I disease, respectively. Extra-nodal involvement was in 16(76.2%), high LDH in 18(85.7%), bone marrow involvement in 8(38.1%) and bulky disease in 5(23.8%) patients. International Prognostic Index (IPI) was 1 in 4(19%), 2 in 9(42.9%), 3 in 8(38.1%) patients (Table-I). Extra-nodal sites were gut in 2(9.1%) while one (4.5%) patient each had the following organs involvement; cervix, gluteal muscle, iliac bone, liver, ovaries, pancreas, parotid gland and testes. Chemotherapy CHOP was given to 16(76.2%), RCHOP 5(23.8%) patients. Prophylactic intrathecal methotrexate was given to 10(47.6%) patients.

**Table-I:** Baseline characteristics of study participants.

*Baseline Characteristics (at time of diagnosis)*	*Mean ± SD or N (%)*
Age (Years)	37.41 ± 10.14
***Gender***	
Male	14 (66.7%)
Female	07 (33.3%)
***Stage of Disease***	
I	3 (14.3%)
II	2 (9.5%)
III	4 (19.0%)
IV	12 (57.1%)
***LDH***	
High	18 (85.7%)
Normal	03 (14.3%)
***Extra Nodal site involvement***	
None	5 (23.8%)
Yes	16 (76.2%)
***Bulky Disease***	
Yes	5 (23.8%)
No	16 (76.2%)
***Bone Marrow Involvement***	
Yes	8 (38.1%)
No	13 (61.9%)
***IPI***	
1	04 (19.0%)
2	09 (42.9%)
3	08 (38.1%)

**Fig.1:**
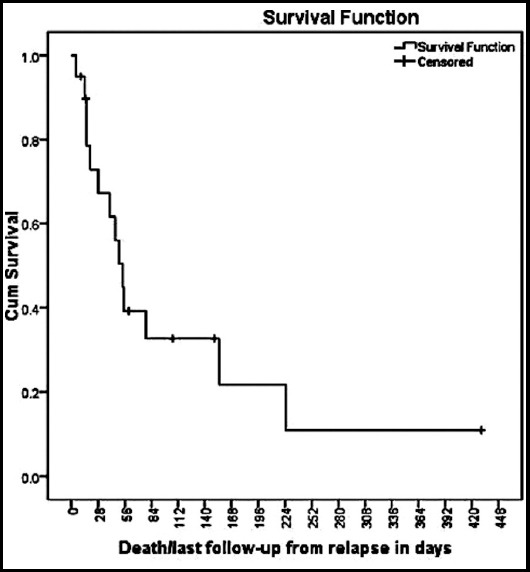


Complete response to chemotherapy was seen in 10 (47.6%), partial response in 3 (14.3%) and disease progression was seen in 8 (38.1%) patients. CNS relapse occurred in 17 (81%) patients within six months after completion of therapy. CNS relapse along with systemic disease was noted in 14(66.6%) patients. Isolated CNS relapse occurred in 7(33.3%) patients. Second line chemotherapy regimens were High Dose Methotrexate5 (23.8%), HDMTX/TRIO IT 3(14.2%), HDMTX/HDAC 2(9.5%), HCVAD 3(14.2%), ICE 4(19.4%), DHAP 1(4.7%), ICE/HDMTX 1(4.7%), none 2(9.5%) (Table-II). Overall median survival of patients with CNS relapse was 54 days (Fig.1). No characteristics were statistically significant when calculated with chi square test. At time of analysis only one patient was alive.

**Table-II:** Subsequent second line chemotherapy.

*Second line Chemotherapy*	*n(%)*
HDMTX	5(23.8%)
High dose methotrexate	
HDMTX/TRIO IT	3(14.2%)
High dose methotrexate/Methoreaxte, Cytrabine, Hydrocortisone	
HDMTX/HDAC	2(9.5%)
High dose methotrexate/High dose Ara-C	
HCVAD Hyperfractionated cylcophospahmide, vincristine, doxorubicin, dexamethasone	3(14.2%)
ICE	4(19.4%)
Ifosphamide, carboplatin, etoposide	
DHAP	1(4.7%)
Dexamethasone, High dose Ara-C, Cisplatin	
ICE/HDMTX	1(4.7%)
Ifosphamide, carboplatin, etoposide/ High dose methotrexate	
None	2(9.5%)
Total	21

## DISCUSSION

In our study, most of the patients were young, advance staged and with high LDH. Only one patient had testicular involvement that is considered as high risk for CNS relapse. Patients who received rituximab along with conventional chemotherapy also developed CNS relapse. In a systematic review it is found that the use of rituximab has not influenced the incidence of CNS relapse compared with the use of CHOP.^20^ However, few reports have shown some benefit of adding rituximab to initial therapy.^21^ Most of the patients developed relapse within six months of the initial treatment that is consistent with published data.^3^-^6^ In this study, isolated CNS relapse (33%) which is higher than the available data.^4^,^22^ Identifying high risk population with aid of prognostic models like CNS-IPI^10^,^11^ and addition of different combinations of CNS directed therapy may help to improve survival in these patients. In our study, majority of patients with CNS relapse had advance stage disease (III/IV), extranodal disease, high LDH and bone marrow involvement, which are considered as high risk population.^9^ About half of patients who had CNS relapse, they had received CNS prophylaxis. This is consistent with established fact that intrathecal MTX may not reduce the risk in all high risk patients. High dose systemic methotrexate and cytarabine had been used as prophylaxis with mixed results.^16^ None of our patient received systemic high dose methotrexate, which has been advocated recently in many studies.^17^,^18^ As there is no current standard validated treatment of CNS relapse^15^, different salvage chemotherapeutic regimens were used according to patient tolerance status. Most of the patients died during second line treatment with very short median survival. Median survival in our study is short compared to available data, which could be due to poor tolerance to salvage chemotherapy in our population and late diagnosis, due to various reasons. If relapse in CNS occurs, then high dose therapy with autologous stem cell transplant can improve survival and this approach has shown better survival in isolated CNS relapse in comparison to CNS relapse with systemic disease.^23^ Our patients with CNS relapse were potential candidate for HDT/ACST but unfortunately, none of these patients could survive to reach that point except one who underwent autologous stem cell transplant on first relapse and allotransplant after second CNS relapse.

## CONCLUSION

Patients with DLBCL who had advanced stage, high LDH and extra-anodal involvement at initial presentation are at high risk for CNS relapse. Once relapsed in CNS, these patients have very poor prognosis. High risk patients may have CNS relapse despite intrathecal chemotherapy prophylaxis.
